# Deletion of the *H240R* Gene in African Swine Fever Virus Partially Reduces Virus Virulence in Swine

**DOI:** 10.3390/v15071477

**Published:** 2023-06-29

**Authors:** Elizabeth Ramirez-Medina, Ayushi Rai, Nallely Espinoza, Alyssa Valladares, Ediane Silva, Lauro Velazquez-Salinas, Manuel V. Borca, Douglas P. Gladue

**Affiliations:** 1 Plum Island Animal Disease Center, ARS, USDA, Greenport, NY 11944, USA; elizabeth.ramirez@usda.gov (E.R.-M.); ayushi.rai@usda.gov (A.R.); nallely.espinoza@usda.gov (N.E.); alyssa.valladares@usda.gov (A.V.); ediane.silva@usda.gov (E.S.); lauro.velazquez@usda.gov (L.V.-S.); 2 Oak Ridge Institute for Science and Education, Oak Ridge, TN 37830, USA

**Keywords:** ASFV, ASF, ASFV *H240R* gene, African swine fever virus, virus virulence, protective immunity

## Abstract

African swine fever (ASF) is a highly contagious disease that affects wild and domestic swine. Currently, the disease is present as a pandemic affecting pork production in Eurasia and the Caribbean region. The etiological agent of ASF is a large, highly complex structural virus (ASFV) harboring a double-stranded genome encoding for more than 160 proteins whose functions, in most cases, have not been experimentally characterized. We show here that deletion of the ASFV gene *H240R* from the genome of the highly virulent ASFV-Georgia2010 (ASFV-G) isolate partially decreases virus virulence when experimentally inoculated in domestic swine. ASFV-G-∆H240R, a recombinant virus harboring the deletion of the *H240R* gene, was produced to evaluate the function of the gene in the development of disease in pigs. While all animals intramuscularly inoculated with 10^2^ HAD_50_ of ASFV-G developed a fatal form of the disease, forty percent of pigs receiving a similar dose of ASFV-G-∆H240R survived the infection, remaining healthy during the 28-day observational period, and the remaining sixty percent developed a protracted but fatal form of the disease compared to that induced by ASFV-G. Additionally, all animals inoculated with ASFV-G-∆H240R presented protracted viremias with reduced virus titers when compared with those found in animals inoculated with ASFV-G. Animals surviving infection with ASFV-G-∆H240R developed a strong virus-specific antibody response and were protected against the challenge of the virulent parental ASFV-G.

## 1. Introduction

African swine fever virus (ASFV) is the causative agent of African swine fever (ASF), a frequently lethal disease affecting domestic pigs and currently damaging the swine production industry in Eurasia. The disease has just been discovered in the Dominican Republic and Haiti after more than 40 years of absence in the Western hemisphere [1]. Currently, no commercial vaccines are available outside of Vietnam, where the first commercially produced vaccines are being used. Therefore, for most countries suffering outbreaks disease control is restricted to culling all infected animals and limiting the mobilization of infected animals.

ASFV is a very large and structurally complex virus, with a large (180–190 kilobase pairs) double-stranded DNA genome encoding more than 150 genes with diverse predicted functions and structures [2,3]. However, the molecular function of many of these genes remains unknown, having only predicted function based on sequence similarities to other viruses or mammalian genes. Development of recombinant ASFV harboring individual gene deletions have been pivotal in understanding gene contribution to viral replication or virulence, resulting in a variety of phenotypes from undetectable or minor changes (i.e., *A859L* [4], *KP177R* [5], *MGF110-1L* [6], *X69R* [7], *MGF360-1L* [8], and *CD2* [9]). In addition, the identification of genes involved in virus virulence in pigs was essential in the rational development of live attenuated ASFV vaccine candidates. Several recombinant ASF vaccine strains have been developed in this way, efficaciously protecting pigs against the challenge of the ASFV-G isolate or its field isolate derivatives [10,11,12,13,14,15]. Thus, the identification of virus genes critical in the process of virulence in domestic pigs is an important initial step in the development of recombinant attenuated vaccine candidates.

Here, we present the characterization of the effect of the deletion of the ASFV gene *H240R* in both the replication ability of the virus in primary swine cultures and its virulence in domestic pigs of ASFV-G. Recently, the *H240R* gene has been shown to strongly inhibit transcription, maturation, and secretion of interleukin-1 [16]. Infection of pigs with a recombinant virus, based on the Chinese field strain HLJ/18 and lacking the *H240R* gene, presented a complete abolishment of the virulence [17].

We demonstrated here that a recombinant virus harboring a deletion of the *H240R* from the genome of virulent ASFV-G isolate (ASFV-G-∆H240R) when compared with the virulent parental ASFV-G, has a similar ability to replicate in swine macrophages cultures but has only a moderate reduction in its virulence when experimentally inoculated in swine.

## 2. Materials and Methods

### 2.1. Viruses and Cells

Production of virus stocks, virus titrations, virus growth curves, and transfection/infections were performed on primary cultures of blood swine macrophages as previously described [18]. In all cases, macrophages were seeded at a density of 5 × 10^6^ cells per ml. The ASFV-G strain is a field isolate kindly provided by Dr. Nino Vepkhvadze, from the Laboratory of the Ministry of Agriculture (LMA) in Tbilisi, Republic of Georgia. Growth curves of the ASFV-G-∆H240R and the parental ASFV-G strain were performed at an MOI of 0.01 HAD_50_ (hemadsorbing doses, as determined in primary swine macrophage cell cultures) as previously described [19]. Sample points were taken at 2, 24, 48, 72, and 96 h post-infection, when cells were frozen at ≤−70 °C, thawed, and the lysates (cell extract and supernatant) titrated by HAD_50_/mL in primary swine macrophage cell cultures in 96-well plates. The presence of the virus-infected cells was assessed by hemadsorption (HA) and virus titers were calculated as previously described [20,21].

### 2.2. Detection of H240R Transcription

The transcriptional profile of the *H240R* gene was evaluated in primary swine macrophage cell cultures infected with ASFV-G using real-time PCR (qPCR). Two well-characterized ASFV genes, the early CP204L (p30) and the late *B646L* (p72) genes, were used as a control for transcription. Porcine macrophage cultures were infected with an MOI of 10 with ASFV-G, and RNA was extracted using an RNeasy Kit (QIAGEN, Hilden, Germany), at 0, 1, 2, 3, 4, 5, 6, 7, 8, and 24 h post-infection. All extracted materials were treated with 2 units of DNase I (BioLabs, Ipswich, MA, USA) and then purified using the Monarch® RNA Cleanup Kit (New England BioLabs, Inc., Ipswich, MA, USA). One ug of RNA was used to produce cDNA using qScript cDNA SuperMix (Quanta bio, Beverly, MA, USA), which was used for the qPCR. Primers and probes for the detection of the *H240L* gene were designed using the ASFV Georgia 2007/1 strain (GenBank Assession # NC_044959.2). Primers forward: 5′GATACTCTTTCGGTCCATGTGG3′, reverse: 5′-CGTTTGCAGGTGTTTATATCCAG 3′and probe: 5′-FAM/TCACCCGTTGTTAGGTTATGGTTTTGGA/MGBNFQ-3′. Primers and probes for the detection of p72, p30, and the β-actin genes were previously described [22]. All qPCRs reactions were performed using the TaqMan Universal PCR Master Mix (Applied Biosystems) under the following amplification conditions: one step at 55 °C for 2 min, followed by one denaturation step at 95 °C for 10 min, then 40 cycles of denaturation at 95 °C for 15 s and annealing/extension at 65 °C for 1 min.

### 2.3. Construction of the ASFV H240R Deletion Mutant

A virus with a deleted *H240R* gene (ASFV-G-∆H240R) was obtained by homologous recombination between the genome of the parental ASFV-G and a recombination transfer vector as previously described [19]. The recombinant transfer vector (p72mCherryΔH240L) contains both flanking genomic regions of the *H240R* gene: the left arm spans between genomic positions 155334-156334 while the right arm is situated between genomic positions 157052-158052 and contains a reporter gene cassette harboring the mCherry fluorescent protein (mCherry) gene under the control of the ASFV p72 late gene promoter [23]. The recombinant transfer vector was obtained by DNA synthesis (Epoch Life Sciences, Sugar Land, TX, USA). As designed, this construction created a 717-nucleotide deletion between nucleotide positions 156335–157051, deleting the majority of the *H240R* ORF sequence and leaving only the last nine nucleotides in the C-terminus that overlap with the C-terminus of ORF R298L. The resulting mutant ASFV-G-∆H240R was purified by consecutive limiting dilution steps based on mCherry activity detection. ASFV-G-∆H240R stock was full-length sequenced using next-generation sequencing (NGS).

### 2.4. Next-Generation Sequencing of ASFV

Virus DNA from the infected macrophage cultures that showed 90–100% CPE was obtained using the Nuclear Extract Kit (Active Motif, Carlsbad, CA, USA). After separation from the nucleus, the cytoplasmic fraction was used to obtain the viral DNA by following the manufacturer’s protocol. Briefly: virus-infected cells were harvested and treated with the hypotonic buffer on ice for 15 min (or until the cell membrane was dissolved). Then, the fraction containing the nucleus was separated by centrifugation, the cytoplasmic fraction was collected, and the DNA was extracted by adding 10% 3M NaOAc by volume to the sample (Sigma-Aldrich, St. Louis, MO, USA) and an equal volume of phenol:chloroform:isoamyl alcohol (25:24:1) with a pH of 6.5–6.9 (Sigma-Aldrich). These were then centrifuged at max speed in a tabletop centrifuge. Then, the aqueous phase was precipitated using 2 volumes of 100% ethanol, washed with the same volume of 70% ethanol, and dried. The obtained pellet of DNA was then resuspended in sterile water. The DNA library was then used for NGS sequencing using a Nextera XT kit in the NextSeq sequencer (Illumnia, San Diego, CA, USA) strictly following the manufacturer’s protocol. Sequence analysis was performed using CLC Genomics Workbench software (CLCBio, Waltham, MA, USA).

### 2.5. Evaluation of ASFV-G-ΔH240R Virulence in Domestic Pigs

The virulence of the recombinant ASFV harboring the *H240R* gene deletion (ASFV-G-∆H240R) was evaluated in 35–40 kg commercial breed pigs. Groups of five pigs were experimentally infected by the intramuscular (IM) route with 10^2^ HAD_50_ of either ASFV-G-∆H240R or the parental virulent ASFV-G strain. The appearance of clinical signs (such as depression, anorexia, staggering gait, purple skin discoloration, diarrhea, and cough), as well as changes in body temperature, were recorded daily throughout the experiment. Blood samples were scheduled to be obtained at days 0, 4, 7, 11, 14, 21, and 28 post-inoculation (pi). The presence of the virus in blood was performed as previously described [4,5,6,7,8,9]. All animal experiments were performed under biosafety level 3 conditions in the animal facilities at Plum Island Animal Disease Center, strictly following a protocol approved by the Institutional Animal Care and Use Committee (225.06-19-R_090716, approved on 9 June 2019).

## 3. Results and Discussion

### 3.1. Evolution of AH240R Gene in Nature

**Figure 1:**
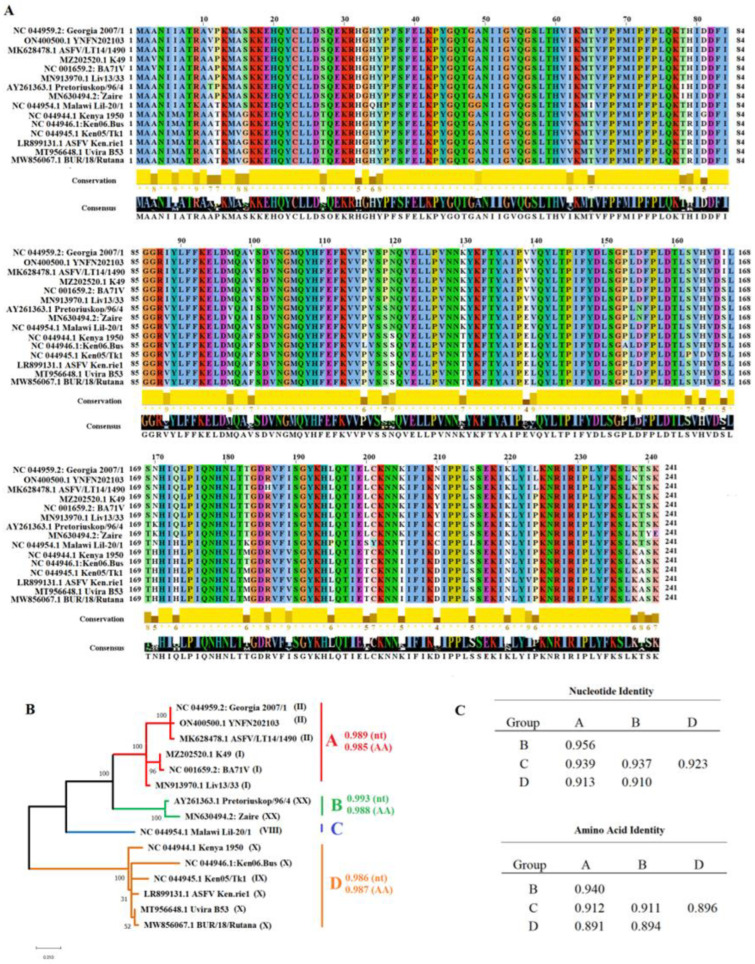
Diversity of *H240R* gene/protein across ASFV isolates. (**A**) Amino acid alignment representing the diversity of the *H240R* protein of ASFV in the field. Residues in white spots represent changes between amino acids with different charges. Conservation plot scores reflect the nature of the change in specific sites: high scores are associated with changes with similar biological properties. Alignment was produced using the software Jalview version 2.11.1.4. (**B**) Phylogenetic analysis was conducted by maximum likelihood, using the Tamura 3-parameter gamma distributed as a model of substitution (AICc score = 3615.336). Based on the full-length sequence of the *H240R* gene, representative ASFV isolates were classified into four groups. Numbers in parentheses represent the genotype classification of different isolates based on the P72 gene. Nucleotide (nt) and Amino acid (AA) values of identity were predicted within diverse groups. (**C**) Pairwise distance analysis using the model p-distance and the bootstrap method to obtain a confidence interval of 95% was conducted to infer the levels of identity between phylogenetic groups predicted during the evolution of the *H240R* gene/protein in nature. Analyses were conducted using the MEGA software version 10.2.5.

To evaluate the evolutionary dynamics of the *H240R* gene of ASFV in nature, we collected a representative group of sequences from the GenBank database. Overall, using the Georgia 2007/1 strain (GenBank access NC_044959.2) as a query, we conducted a Blast analysis to obtain a total of 15 viral sequences that represented the nucleotide diversity of the *H240R* gene in nature (Figure 1). Initial pairwise analysis and distance analysis using the p-distance model and the bootstrap method (*p* < 0.05) [24], revealed a nucleotide identity between 99.86% and 89.94% (~94.19%) and an amino acid identity between 99.58% and 87.96% (~92.63%), showing the high conservation of *H240R* protein among ASFV in nature (Figure 1A). Three different phenotypes of this protein were found among isolates associated with the Eurasian pandemic lineage (genotype II). One was represented by the Georgia 2007/1 strain, which constitutes the most prevalent phenotype among 100 ASFV isolates reported in GenBank Database. A second, less prevalent phenotype associated with the isolate ASFV/LT14/1490 (isolated from a pig in Lithuania in 2014), which presents a single amino acid replacement *R186H*, was observed in two distinct isolates: ASFV/POL/2015/Podlaskie (isolated from a pig in Poland 2015) and ASFV/Ulyanovsk 19/WB-5699 (isolated from a wild boar in Russia 2019). Finally, a unique phenotype including the K238N replacement was recorded in the isolate YNFN202103 (isolated from a pig in China in 2021). The overall results indicate that the *H240R* gene has been subject to diversification during the current pandemic outbreak of ASFV. Interestingly, specific amino acid replacements at positions 167, 210, and 224 appeared putative of the Eurasian lineage (Figure 1A), indicating the *H240R* gene can serve as a marker for molecular epidemiology studies to discriminate the presence of the pandemic lineage with other ASFV.

Phylogenetic analysis conducted on the *H240R* gene presented the existence of at least four distinct genetic groups. Within each group, high levels of identity at both nucleotide and amino acid levels were predicted (Figure 1B). However, when levels of identity were contrasted between groups, important differences were observed (Figure 1C), suggesting the existence of potential functional differences among diverse phenotypes of *H240R* protein in nature. The above can be exemplified if we consider the decreased levels of amino acid identity between viruses, including in groups A and D (Figure 1C). The above may be particularly relevant if we take into account the results presented in our study regarding the role of virulence in pigs of the *H240R* gene. Future studies are needed to experimentally confirm the potential functional differences among *H240R* phenotypes present in nature.

Overall, no homology with other protein families was predicted when the amino acid sequence of the Georgia 2007/1 ASFV strain was evaluated using the software InterPro version 93.0. This software concentrates the information from 13 protein databases, making this tool a reliable resource to identify protein families, functional sites, and domains [25]. To acquire further insights into the potential relevance of some codon sites during the evolution of the *H240R* gene, we conducted a phylogenomic analysis using a previously described strategy [26]. First, to learn about the predominant evolutionary force during the evolution of the *H240R* gene in nature, this gene was evaluated using the algorithm fixed effects likelihood (FEL) [27] Overall dN/dS ratio equal to 0.365 indicates that purifying selection is the dominant force shaping the evolution of *H240R* gene in nature. This ratio was similar to the one identified in other genes with unknown functions in ASFV (*A137R*, *E184L,* and *H108R*). Interestingly, in all these cases, the deletion of these genes resulted in the reduction of virulence in pigs [14,28,29]. More studies are needed to understand the importance of this finding in the evolution of ASFV.

**Figure 2:**
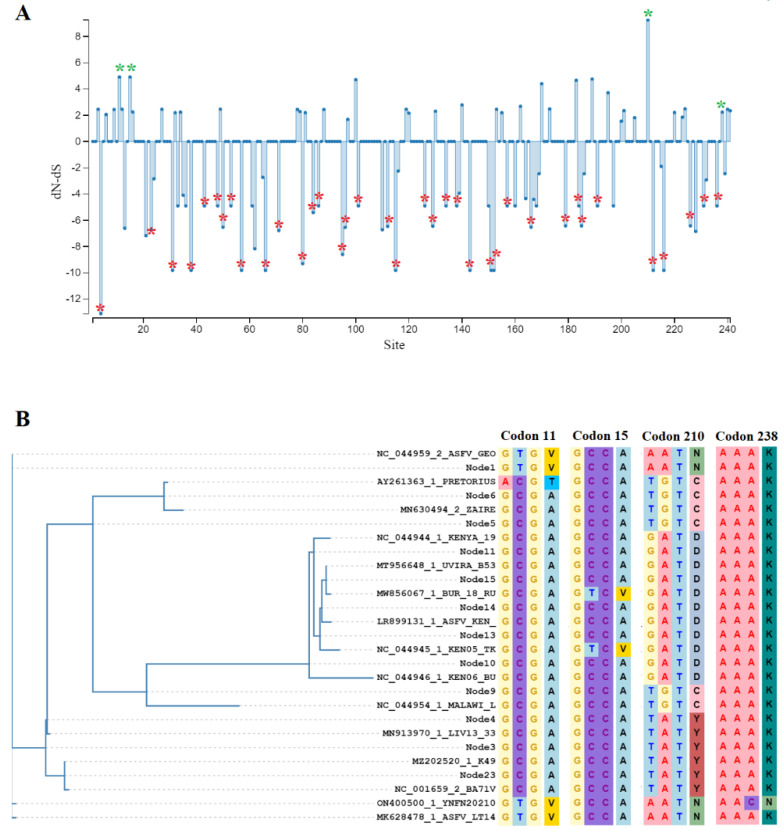
Evolutionary dynamics of *H240R* gene in nature. (**A**) The graphic represents the dN (rate of evolution at non-synonymous sites) and dS (rate of evolution at synonymous sites) ratio (dN-dS) at specific codon sites in the *H240R* gene of ASFV. Green asterisks represent codons detected under positive selection by the algorithms MEME and FUBAR (cutoff values of *p* = 0.1 and posterior probability = 0.9, respectively). Red asterisks represent codons detected by FEL (cutoff value of *p* = 0.1) under negative selection (codon sites: 4, 23, 31, 38, 43, 48, 50, 53, 57, 66, 71, 80, 84, 86, 95, 96, 101, 112, 115, 126, 129, 134, 138, 143, 151, 152, 157, 166, 179, 184, 185, 191, 212, 216, 226, 231, and 236). The amino acids encoded by these codons can be visualized in Figure 1A. (**B**) Ancestral reconstruction conducted by the algorithm Single Likelihood Ancestor Counting (SALC) [14], representing the codons predicted under positive selection by MEME and FUBAR analyses.

Overall, a total of 37 codon sites distributed along the *H240R* gene were identified under negative selection (Figure 2A). The high conservation of these codons during the evolution of the *H240R* gene strongly suggests the potential significance of these sites in the function of the *H240R* protein. Furthermore, the preservation of these sites during the evolution of the *H240R* gene takes more relevance considering the presence of early ASFV isolates such as K49 and Kenya 1950 (collected from pigs in the Democratic Republic of Congo and Kenya in 1949 and 1950 respectively), indicating that these sites have been preserved for at least 70 years. Based on the relevance of *H240R* in virulence in pigs, the identification of these 37 sites also represents a potential framework for future research intended to identify critical functional sites in the *H240R* protein.

On the other hand, using the algorithms mixed effects model of evolution (MEME) [30], and Fast Unconstrained Bayesian AppRoximation (FUBAR) [31] we identified codon sites 11, 15, 210, and 238 as positively selected sites (Figure 2A). In this context, sites 11, 15, and 238 appeared to be associated with evolutionary events of episodic selection impacting isolates at specific genetic groups (Figure 2B). Conversely, the selection at site 210 represents an event of pervasive selection, affecting isolates in all genetic groups (Figure 2B). Future experimental research is needed to confirm the relevance of these sites in the function of the H240R protein. Finally, no evidence of recombination in the *H240R* gene was detected by the genetic algorithm for recombination detection (GARD) [32].

### 3.2. Detection of H240R Transcription

**Figure 3:**
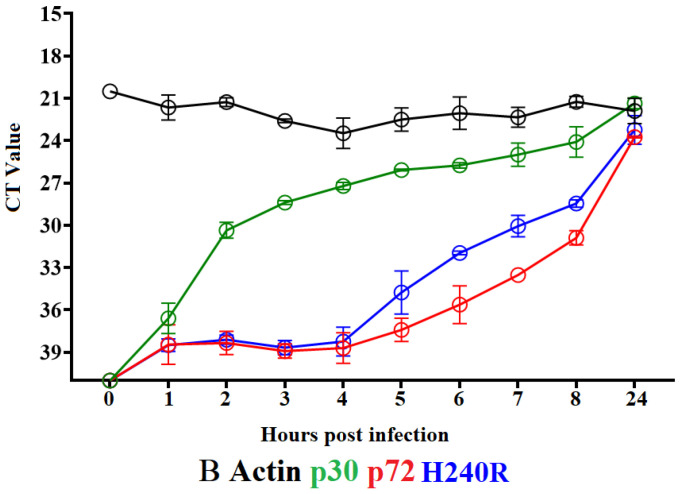
Expression profile of *H240R* gene of ASFV during *in vitro* infection of porcine macrophages. Reverse transcription followed by qPCR was used to evaluate the expression profile of the *H240R* gene during *in vitro* infection at different time points up to 24 h. As a reference for this analysis, we used qPCRs to specifically detect the expression of genes encoding ASFV proteins p30 (early expression) and p72 (late expression). Additionally, the actin gene was used as a control to evaluate the quality and levels of RNA during the infection at different time points.

To determine the kinetics of transcription of the *H240R* gene during the replication cycle of ASFV, a time course experiment was performed in primary swine macrophages infected with ASFV-G. Swine macrophage cultures were infected (MOI = 1) with ASFV-G, and samples of cell lysates were taken at 0, 4, 6, 8, and 24 hpi. The presence of H240R RNA was evaluated with the two-step RT-PCR described in the Material and Methods section. Transcription of H240R was first detected after 5 hpi and progressively increased until 24 hpi (Figure 3). The kinetic expression of the two well-characterized ASFV genes, the early gene encoding for p30 (*CP204L*) and the late gene encoding for p72 (*B646L*) were included as a reference of early and late transcription profiles, respectively. Results demonstrated that the expression of *H240R* overlaps that of the *B646L* gene, suggesting that it encodes for a late protein.

### 3.3. Development of the ASFV-G-ΔH240R Deletion Mutant

**Figure 4:**
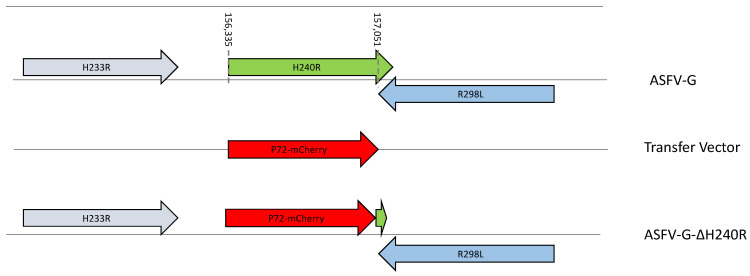
Schematic for the development of ASFV-G-∆H240R. The transfer vector contains the p72 promoter and a mCherry cassette. The gene positions are indicated. The homologous arms were designed to have flanking ends on both sides of the deletion/insertion cassette. The nucleotide positions of the area that was deleted in the ASFV-G genome are indicated by the dashed lines. The resulting ASFV-G-∆H240R virus with the cassette inserted is shown at the bottom.

The high level of nucleotide and amino acid conservation of the *H240R* gene among different ASFV isolates, along with its function as a modulator of the IL-1 response [33] suggest that *H240R* may play an important role in the process of virus replication during the infection *in vitro* and *in vivo*. To assess the potential critical role of *H240R* during the process of virus replication in swine macrophages and in infected animals, a recombinant virus with the *H240R* gene deleted was developed (ASFV-G-∆H240R) using ASFV-G as the parental virus. The *H240R* gene was deleted by replacing 239 amino acid residues of the *H240R* ORF with the p72mCherry cassette by homologous recombination [12]. An area covering 717 bp between nucleotide positions 156335-157051 was eliminated from the genome of ASFV-G, deleting the majority of the *H240R* gene and leaving only the last 9 bp of the C-terminus so as not to disturb the C-terminus of *R298L* which has an overlapping ORF. This deletion was then substituted with a 1226-bp cassette containing the p72mCherry construct (see Material and Methods) (Figure 4). The recombinant ASFV-G-∆H240R stock was purified after successive limiting dilution steps using primary swine macrophage cell cultures.

To evaluate the accuracy of the alterations induced into the ASFV-G-∆H240R genome, the full genome sequence was obtained by NGS using an Illumina NextSeq^®^ 500. A total of 2,640,928 reads were aligned to the ASFV genome. A comparative study between genomes of ASFV-G-∆H240R, and ASFV-G demonstrates a deletion of 717 nucleotides and an insertion of 1226 nucleotides corresponding to the p72-mCherry cassette sequence. No undesired additional changes were detected as a result of the process of production and purification of ASFV-G-∆H240R. In addition, NGS data showed the absence of the parental ASFV-G genome as a potential contaminant in the ASFV-G-∆H240R stock.

### 3.4. Assessment of Replication of ASFV-G-∆H240R in Swine Macrophages Cultures

**Figure 5:**
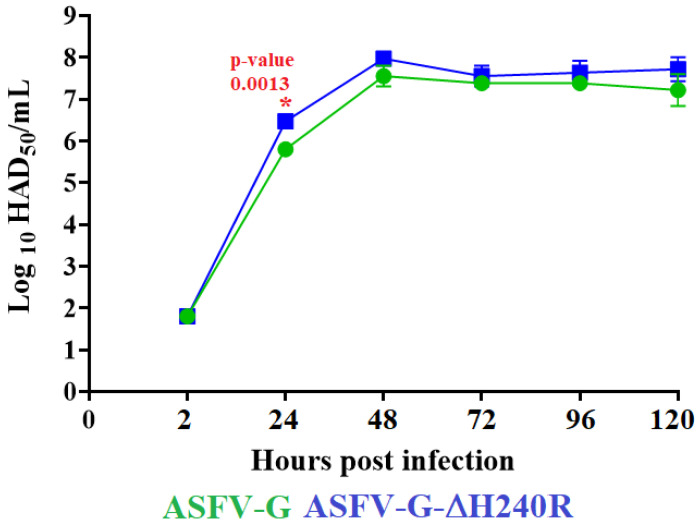
*In vitro* growth kinetics in primary swine macrophage cell cultures for ASFV-G-∆H240R and parental ASFV-G (MOI = 0.01). Samples were taken from two independent experiments at the indicated time points and titrated. The data represent the means and standard deviations of three replicas. Sensitivity using this methodology for detecting the virus is ≥log10 1.8 HAD_50_/mL. (*) Indicates significant differences.

To assess the role of the *H240R* gene in the course of virus replication, the capacity of the recombinant ASFV-G-∆H240R to replicate in primary swine macrophage cultures was evaluated and compared to that of the parental ASFV-G using a multistep growth curve. Macrophage cultures were infected (MOI of 0.01) with either ASFV-G-∆H240R or ASFV-G and virus yields were quantified at 2, 24, 48, 72, and 96 h post-infection. The results showed that ASFV-G-∆H240R presents similar replication kinetics to the parental ASFV-G. A statistical difference was found in virus titers at 24 h pi, but cannot be considered of biological significance (Figure 5). Therefore, it appears that deletion of the *H240R* gene from the ASFV-G genome does not affect final virus replication in primary swine macrophage cultures. These results appear different from those obtained by deleting the *H240R* gene from the highly virulent Chinese field isolate HLJ/18 [16]. Those reports showed that the HLJ/18 virus lacking the *H240R* gene has a decreased ability to replicate in pulmonary-derived swine macrophages with a reduction in virus yield between 10 and 100 folds, compared with the parental field isolate. Since MOI and sampling time points are similar in the experiments reported using the HLJ/18 [16] and ours using the ASFV-G strain, differences in the results should be due to genetic differences inherent to each of the field isolates.

### 3.5. Assessment of ASFV-G-∆H240R Virulence in Swine

**Figure 6:**
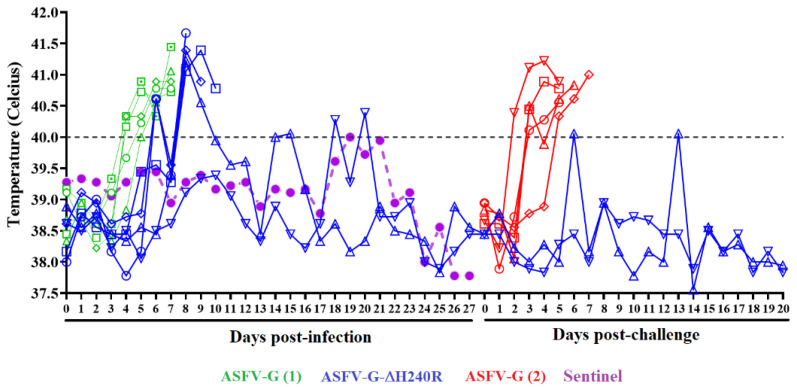
Progression of body temperature in animals (5 animals/group) IM inoculated with 10^2^ HAD_50_ of either ASFV-G-∆H240R or parental ASFV-G.

**Figure 7:**
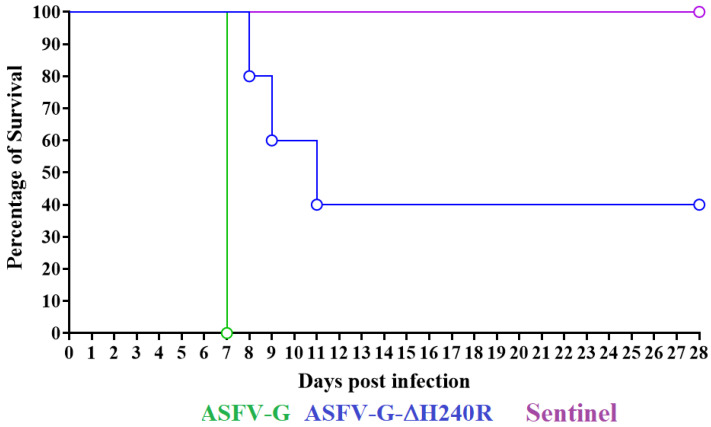
Progression of mortality in animals (5 animals/group) IM infected with 10^2^ HAD_50_ of either ASFV-G-∆H240R or parental ASFV-G.

The assessment of the potential role of the *H240R* gene in ASFV-G virulence was performed by the IM inoculation of ASFV-G-∆H240R in a group of five 35–40 kg domestic pigs at a dose of 10^2^ HAD_50_. The evolution of the appearance of clinical signs associated with ASF was monitored daily for 28 days and compared with the effect of the inoculation of parental virulent ASFV-G, administered by the same route and dose to a control group of pigs. A sentinel animal was included cohabitating with the ASFV-G-∆H240R inoculated animals to assess virus transmission. As expected, all animals inoculated with the parental ASFV-G presented an increase in body temperature (over 40 °C) on day 4–5 day evolving to full clinical disease (anorexia, depression, diarrhea, staggering gait, and purple skin discoloration) with all animals euthanized by day 7 pi due to the severity of the clinical signs (Figure 6 and Figure 7).

Animals inoculated with ASFV-G-∆H240R present quite heterogeneous behavior. Four of the animals presented a sudden rise in body temperature (over 40 °C) by day 7 pi (2 to 3 days after the fever onset in animals inoculated with the parental virus). The clinical status of three of these pigs evolved to a more serious form of the disease and were euthanized due to the severity of the clinical disease by days 8, 9, and 10 pi, respectively. The fourth remaining animal, which presented peak fever at day 8 pi, returned to normal body temperature values by day 10 pi, presenting a transitory and modest peak of fever by day 14 and 15 pi and remaining within normal values until the end of the observational period (28 days pi). This animal did not present any clinical signs associated with ASF with the exception of transient mild depression and anorexia between days 7–9 pi. The fifth animal inoculated with ASFV-G-∆H240R survived the infection without showing any clinical sign of the disease except for occasional peaks of mild fever by days 18 and 20 pi (Figure 6 and Figure 7). The sentinel animal remained clinically normal during the 28-day observational period with body temperature readings oscillating within normal values (Figure 6).

The genetic characteristics of the infectious virus present in the specimens at the time of euthanasia were assessed in order to ensure that the virulent phenotype observed in the ASFV-G-∆H240R inoculated animals was due to the recombinant virus and not a result of a potential presence of the virulent parental virus contaminating the ASFV-G-∆H240R inoculum. The genome of viruses isolated from all five animals in this group was subject to full-length sequencing by NGS, and in all the cases results demonstrated that the isolated virus was ASFV-G-∆H240R.

Therefore, it appears that deletion of the *H240R* gene produces just a moderate decrease in the virulence of the ASFV-G isolate, represented by the presence of a lethal but protracted form of the disease in 3 out 5 animals and survival with or without the presence of a transient clinical disease in the other two animals. We have observed this heterogenous pattern of disease kinetics while testing other recombinant viruses harboring a deletion of specific virus genes [23,29,34].

These results differ from those reported for the HLJ/18 virus lacking the *H240R* gene [17]. That report showed that animals IM inoculated with 10^3^ HAD_50_ all survived the infection without showing any clinical sign of the disease except for a transient and mild elevation of body temperature by day 8 pi. Even doses as high as 10^5^ HAD_50_ only produce a protracted clinical disease in 2 out 5 inoculated animals, with only one of the two animals developing disease euthanized by day 16 pi due to the severity of the clinical signs. The reasons behind the striking differences between results reported with the HLJ/18 strain and ours are not clear but indicate that the role of the *H240R* gene in disease production in domestic swine is highly dependent on the genetic background of the ASFV isolate considered. It is worth indicating here that animals inoculated with 10^3^ HAD_50_ of HLJ/18 appear to develop a more protracted clinical disease than those inoculated with 10^2^ HAD_50_ of ASFV-G since they were euthanized by day 10 pi [17] while those receiving a lower dose of ASFV-G were euthanized by day 7 pi. It could be possible that HLJ/18 isolate is slightly less virulent than ASFV-G and because of that, the deletion of the *H240R* gene causes a stronger attenuation in HLJ/18 than in ASFV-G. The fact that deletion of a particular gene in different ASFV isolates produces different alterations in the virulent phenotype has already been reported. Deletion of different virus genes, even with some of them highly conserved across ASFV isolates, have been reported to produce different effects of virus virulence in domestic swine: *9GL* [22,35], *NL* [36,37], and *CD2* [9,38]. Our results indicate that *H240R* is another of these genes that have a differential contribution to virus virulence depending on the genetic background of the virus isolate being taken into consideration. [39].

**Figure 8:**
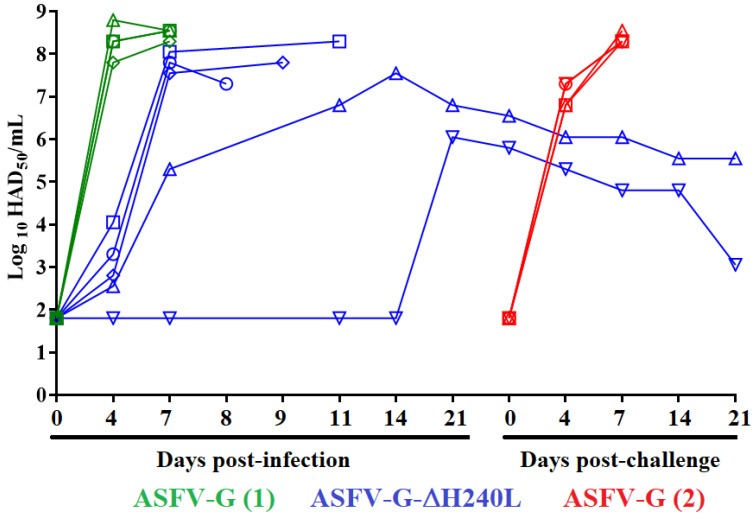
Viremia titers detected in pigs IM inoculated with 10^2^ HAD_50_ of either ASFV-G-∆H240L, or ASFV-G, as determined in primary swine macrophages. Each symbol represents the data of individual animals in each of the groups. Sensitivity of virus detection: ≥log10 1.8 TCID_50_/mL.

The ability of recombinant ASFV-G-∆H240R to replicate during the experimental infection in pigs was evaluated by assessing viremia titers throughout the 28-day experimental period and comparing them to those observed in animals inoculated with parental ASFV-G. Viremia titers in animals experimentally inoculated with parental ASFV-G presented high titers (ranging from 10^7.55^–10^8.55^ HAD_50_/mL) at day 4 pi, remaining high until day 7 pi when all animals were euthanized (Figure 8). Viremias in animals inoculated with ASFV-G-∆H240R presented a heterogeneous pattern in accordance with the presentation of their clinical evolution. The three animals that developed a lethal form of the disease presented low virus titers (10^2.33^–10^4.33^HAD_50_/mL) by day 4 pi, reaching very high viremia values (10^7.55^–10^8.33^ HAD_50_/mL) by day 7 pi, remaining high until the day they needed to be euthanized due to the severity of the clinical disease. The fourth animal presenting an early onset of clinical disease, but which ultimately survived the observation period, presented low viremia titers (10^2.33^ HAD_50_/mL) at day 4 pi, reaching intermediate values (10^5.33^ HAD_50_/mL) by day 7 pi fluctuating between values of 10^7.33^ to 10^6.55^ HAD_50_/mL) until the end of the experimental period. The fifth animal, which did not show clinical signs associated with the disease, showed no detectable viremia titers (equal or less than 10^1.80^ HAD_50_/mL) until day 21st pi (when reached values of (10^5.66^ HAD_50_/mL) remaining at similar or lower values at the end of the observational period (28 days pi). Animals inoculated with HLJ/18 virus lacking the *H240R* gene [17] all presented moderate viremias that lasted until the end of the experiment (21 days pi).

**Figure 9:**
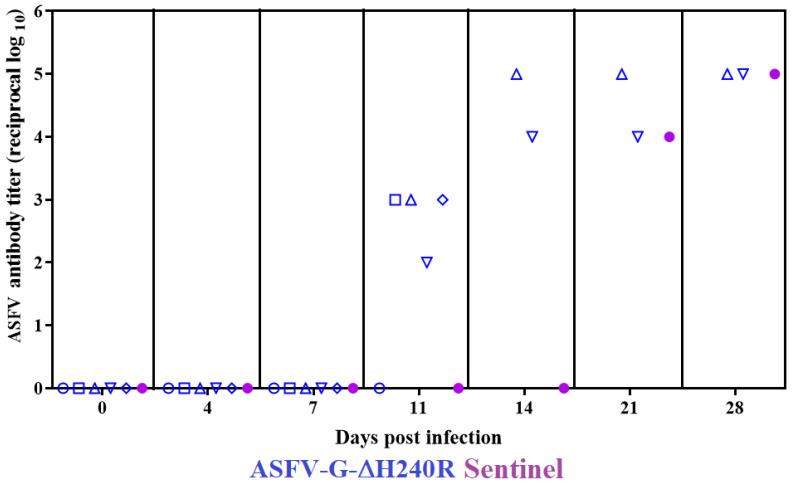
ASFV-specific antibody titers detected in pigs IM inoculated with 10^2^ HAD_50_ of ASFV-G-∆H240R. Each symbol represents the data of individual animals in each of the groups.

Most animals surviving infection with a virulent strain of ASFV developed a serologic antibody response to the virus. The presence of ASFV-specific antibodies in the serum of animals inoculated with ASFV-G-∆H240R was evaluated with an in-house developed ELISA [10]. We observed that the animal euthanized by day 11 pi developed an incipient antibody response and the two surviving animals developed a robust antibody reaction starting by day 11 pi and reaching maximum levels by day 28 pi (Figure 9). Interestingly, the sentinel animal, though it did not show a systemic virus infection (Figure 8), developed a late but strong antibody response reaching levels as high as the inoculated animals (Figure 9).

Most of the pigs resisting an ASFV infection are protected against the challenge of the virulent parental strain [10,12,13,14,39,40,41]. To evaluate if pigs inoculated with ASFV-G-∆H240R survive the infection with the highly virulent ASFV-G at day 28 days after ASFV-G-∆H240R infection, the two surviving pigs were challenged by IM with 10^2^ HAD_50_ of ASFV-G along with 5 control naïve animals who were similarly challenged. While the all-control pigs developed a lethal form of ASF, being euthanized between day 6 and 8 post-challenge, the two pigs previously inoculated with ASFV-G-∆H240R remained completely asymptomatic during the entire observational period (21 days post-challenge).

The viremia titers in challenged animals followed the evolution of their clinical signs. Viremia titers in the control group were high (ranging between 10^6.5^–10^7.5^ HAD_50_/mL) at day 4 pi, and still higher (around 10^8^ HAD_50_/mL) when animals were euthanized due to the severity of the clinical disease (Figure 8). Conversely, viremia titers in the ASFV-G-∆H240R inoculated animals gradually declined from the time of challenge to the end of the observational period. Therefore, animals surviving the infection with ASFV-G-∆H240R were protected against the challenge of the virulent parental virus.

The results reported here demonstrate that the deletion of the *H240R* gene in the context of the ASFV-G isolate, though it does not affect virus replication in the natural target cell, swine macrophages, is moderately involved in the process of virus virulence. Our results demonstrated that animals inoculated with relatively low doses of the virus (10^2^ HAD_50_/mL) produced a lethal, though protracted, clinical form of the disease in 60% of the inoculated animals, with a 40% survival after recovering from a mild clinical disease. Our results differ from those recently published using a Chinese virulent field strain [17] where animals inoculated with higher doses (10^3^ and 10^5^ HAD_50_/mL) of a virus lacking the *H240R* gene did not induce clinical disease with the exception of one animal receiving 10^5^ HAD_50_/mL. Differences in the effect of the deletion of a particular gene in ASFV virulence have been already reported for several genes. Deletion of different virus genes, even those highly conserved across ASFV isolates, have been reported to produce different effects of virus virulence in domestic swine even in those that are highly conserved across different isolates [42]. This interpretation relays that potential differences in the genetic background of each of the different field isolates may affect the outcome of the effect of the deletion of a particular viral gene. Genomic differences have been reported in comparative studies based on full-length genomic sequences between ASFV isolates ASFV-G and HLJ/18 [43] and perhaps are responsible for the different effects of the deletion of the *H240R* gene in each of them. Further complex studies will be necessary in order to prove this hypothesis. An alternative explanation would be that the molecular design to delete the *H240R* gene may cause alterations in the transcriptional activity of genes flanking the *H240R* gene. As explained earlier, our construct does not completely delete the *H240R* gene so as not to potentially affect the transcription of the partially overlapping gene *R298L* located at the C-terminus of the *H240R* gene. Our interpretation of the methodologies used in the development of the recombinant virus HLJ/18 lacking the *H240R* gene [17] is that transcription of the adjacent *R298L* may be affected. The protein encoded by *R298L* has been shown to be part of the virus particle and, although the role of the ASFV gene *R298L* in virus replication or virulence in pigs has not been experimentally assessed, it has been predicted to function as a serine/threonine protein kynase 1 [33]. Therefore, the *R298L* function may be important in some virus functions, including virulence I domestic swine.

The attenuation of ASFV virulence by deletion of a single gene is not a very common feature. Only seven individual genes have been reported to produce full attenuation of ASFV-G (or its derivative isolates) virulence: *I177L* [10,44], *9GL* [35], *A137* [14], *I226R* [45], *I267L* [46], *MGF110-9L* [39] and *MGF505-7R* [47].

It is interesting to mention the fact that while the sentinel animal did not show detectable virus in its blood during the observational period, it did develop virus-specific antibodies indicating the presence of virus shedding from the ASFV-G-∆H240R infected animals.

In summary, we determined that *H240R* is a non-essential gene since its deletion from the ASFV-G genome does not significantly alter virus replication in swine macrophage cultures and its deletion from the ASFV-G isolate causes a very moderate decrease in virus virulence in domestic pigs, in clear contrast to previous reported results indicating a critical role for the *H240R* gene in virus virulence.

Identification of ASFV genes involved in virulence has been shown as essential in the development of attenuated virus strains. Therefore, their correct characterization and genetic manipulation are the first step in designing and producing recombinant ASF live attenuated vaccine candidates.

## Data Availability

All data is included in the manuscript.

## References

[1] Gonzales W., Moreno C., Duran U., Henao N., Bencosme M., Lora P., Reyes R., Nunez R., De Gracia A., Perez A.M. (2021). African swine fever in the Dominican Republic. Transbound. Emerg. Dis..

[2] Tulman E.R., Delhon G.A., Ku B.K., Rock D.L., Etten V. (2009). African Swine Fever Virus In Lesser Known Large dsDNA Viruses.

[3] Spinard E., Azzinaro P., Rai A., Espinoza N., Ramirez-Medina E., Valladares A., Borca M.V., Gladue D.P. (2022). Complete Structural Predictions of the Proteome of African Swine Fever Virus Strain Georgia 2007. Microbiol. Resour. Announc..

[4] Ramirez-Medina E., Vuono E.A., Pruitt S., Rai A., Espinoza N., Velazquez-Salinas L., Gladue D.P., Borca M.V. (2021). Evaluation of an ASFV RNA Helicase Gene A859L for Virus Replication and Swine Virulence. Viruses.

[5] Vuono E.A., Ramirez-Medina E., Pruitt S., Rai A., Espinoza N., Velazquez-Salinas L., Gladue D.P., Borca M.V. (2021). Evaluation of the Function of the ASFV KP177R Gene, Encoding for Structural Protein p22, in the Process of Virus Replication and in Swine Virulence. Viruses.

[6] Ramirez-Medina E., Vuono E., Pruitt S., Rai A., Silva E., Espinoza N., Zhu J., Velazquez-Salinas L., Borca M.V., Gladue D.P. (2021). Development and *In Vivo* Evaluation of a MGF110-1L Deletion Mutant in African Swine Fever Strain Georgia. Viruses.

[7] Ramirez-Medina E., Vuono E., Pruitt S., Rai A., Silva E., Zhu J., Velazquez-Salinas L., Gladue D.P., Borca M.V. (2020). X69R Is a Non-Essential Gene That, When Deleted from African Swine Fever, Does Not Affect Virulence in Swine. Viruses.

[8] Ramirez-Medina E., Vuono E.A., Rai A., Pruitt S., Silva E., Velazquez-Salinas L., Zhu J., Gladue D.P., Borca M.V. (2020). Evaluation in Swine of a Recombinant African Swine Fever Virus Lacking the MGF-360-1L Gene. Viruses.

[9] Borca M.V., O’Donnell V., Holinka L.G., Risatti G.R., Ramirez-Medina E., Vuono E.A., Shi J., Pruitt S., Rai A., Silva E. (2020). Deletion of CD2-like gene from the genome of African swine fever virus strain Georgia does not attenuate virulence in swine. Sci. Rep..

[10] Borca M.V., Ramirez-Medina E., Silva E., Vuono E., Rai A., Pruitt S., Holinka L.G., Velazquez-Salinas L., Zhu J., Gladue D.P. (2020). Development of a Highly Effective African Swine Fever Virus Vaccine by Deletion of the I177L Gene Results in Sterile Immunity against the Current Epidemic Eurasia Strain. J. Virol..

[11] Tran X.H., Le T.T.P., Nguyen Q.H., Do T.T., Nguyen V.D., Gay C.G., Borca M.V., Gladue D.P. (2021). African swine fever virus vaccine candidate ASFV-G-DeltaI177L efficiently protects European and native pig breeds against circulating Vietnamese field strain. Transbound. Emerg. Dis..

[12] O’Donnell V., Risatti G.R., Holinka L.G., Krug P.W., Carlson J., Velazquez-Salinas L., Azzinaro P.A., Gladue D.P., Borca M.V. (2017). Simultaneous deletion of the 9GL and UK genes from the African swine fever virus Georgia 2007 isolate offers increased safety and protection against homologous challenge. J. Virol..

[13] O’Donnell V., Holinka L.G., Gladue D.P., Sanford B., Krug P.W., Lu X., Arzt J., Reese B., Carrillo C., Risatti G.R. (2015). African swine fever virus Georgia isolate harboring deletions of MGF360 and MGF505 genes is attenuated in swine and confers protection against challenge with virulent parental virus. J. Virol..

[14] Gladue D.P., Ramirez-Medina E., Vuono E., Silva E., Rai A., Pruitt S., Espinoza N., Velazquez-Salinas L., Borca M.V. (2021). Deletion of the A137R Gene from the Pandemic Strain of African Swine Fever Virus Attenuates the Strain and Offers Protection against the Virulent Pandemic Virus. J. Virol..

[15] Teklue T., Wang T., Luo Y., Hu R., Sun Y., Qiu H.J. (2020). Generation and Evaluation of an African Swine Fever Virus Mutant with Deletion of the CD2v and UK Genes. Vaccines.

[16] Zhou P., Li L.F., Zhang K., Wang B., Tang L., Li M., Wang T., Sun Y., Li S., Qiu H.J. (2022). Deletion of the H240R Gene of African Swine Fever Virus Decreases Infectious Progeny Virus Production Due to Aberrant Virion Morphogenesis and Enhances Inflammatory Cytokine Expression in Porcine Macrophages. J. Virol..

[17] Huang L., Liu H., Ye G., Liu X., Chen W., Wang Z., Zhao D., Zhang Z., Feng C., Hu L. (2023). Deletion of African Swine Fever Virus (ASFV) H240R Gene Attenuates the Virulence of ASFV by Enhancing NLRP3-Mediated Inflammatory Responses. J. Virol..

[18] Borca M.V., Berggren K.A., Ramirez-Medina E., Vuono E.A., Gladue D.P. (2018). CRISPR/Cas Gene Editing of a Large DNA Virus: African Swine Fever Virus. Bio-Protocol..

[19] Borca M.V., O’Donnell V., Holinka L.G., Sanford B., Azzinaro P.A., Risatti G.R., Gladue D.P. (2017). Development of a fluorescent ASFV strain that retains the ability to cause disease in swine. Sci. Rep..

[20] Reed L.J.M.H. (1938). A simple method of estimating fifty percent endpoints. Am. J. Hyg..

[21] Borca M.V., Holinka L.G., Berggren K.A., Gladue D.P. (2018). CRISPR-Cas9, a tool to efficiently increase the development of recombinant African swine fever viruses. Sci. Rep..

[22] O’Donnell V., Holinka L.G., Krug P.W., Gladue D.P., Carlson J., Sanford B., Alfano M., Kramer E., Lu Z., Arzt J. (2015). African swine fever virus Georgia 2007 with a deletion of virulence-associated gene 9GL (B119L), when administered at low doses, leads to virus attenuation in swine and induces an effective protection against homologous challenge. J. Virol..

[23] Ramirez-Medina E., Vuono E.A., Pruitt S., Rai A., Espinoza N., Valladares A., Silva E., Velazquez-Salinas L., Borca M.V., Gladue D.P. (2022). Deletion of African Swine Fever Virus Histone-like Protein, A104R from the Georgia Isolate Drastically Reduces Virus Virulence in Domestic Pigs. Viruses.

[24] Kumar S., Stecher G., Li M., Knyaz C., Tamura K. (2018). MEGA X: Molecular Evolutionary Genetics Analysis across Computing Platforms. Mol. Biol. Evol..

[25] Paysan-Lafosse T., Blum M., Chuguransky S., Grego T., Pinto B.L., Salazar G.A., Bileschi M.L., Bork P., Bridge A., Colwell L. (2023). InterPro in 2022. Nucleic Acids Res..

[26] Velazquez-Salinas L., Zarate S., Eberl S., Gladue D.P., Novella I., Borca M.V. (2020). Positive Selection of ORF1ab, ORF3a, and ORF8 Genes Drives the Early Evolutionary Trends of SARS-CoV-2 During the 2020 COVID-19 Pandemic. Front. Microbiol..

[27] Kosakovsky Pond S.L., Frost S.D. (2005). Not so different after all: A comparison of methods for detecting amino acid sites under selection. Mol. Biol. Evol..

[28] Ramirez-Medina E., Vuono E., Rai A., Pruitt S., Espinoza N., Velazquez-Salinas L., Pina-Pedrero S., Zhu J., Rodriguez F., Borca M.V. (2022). Deletion of E184L, a Putative DIVA Target from the Pandemic Strain of African Swine Fever Virus, Produces a Reduction in Virulence and Protection against Virulent Challenge. J. Virol..

[29] Vuono E., Ramirez-Medina E., Silva E., Rai A., Pruitt S., Espinoza N., Valladares A., Velazquez-Salinas L., Gladue D.P., Borca M.V. (2022). Deletion of the H108R Gene Reduces Virulence of the Pandemic Eurasia Strain of African Swine Fever Virus with Surviving Animals Being Protected against Virulent Challenge. J. Virol..

[30] Murrell B., Wertheim J.O., Moola S., Weighill T., Scheffler K., Kosakovsky Pond S.L. (2012). Detecting individual sites subject to episodic diversifying selection. PLoS Genet..

[31] Murrell B., Moola S., Mabona A., Weighill T., Sheward D., Kosakovsky Pond S.L., Scheffler K. (2013). FUBAR: A fast, unconstrained bayesian approximation for inferring selection. Mol. Biol. Evol..

[32] Kosakovsky Pond S.L., Posada D., Gravenor M.B., Woelk C.H., Frost S.D. (2006). GARD: A genetic algorithm for recombination detection. Bioinformatics.

[33] Alejo A., Matamoros T., Guerra M., Andres G. (2018). A Proteomic Atlas of the African Swine Fever Virus Particle. J. Virol..

[34] Ramirez-Medina E., Vuono E., Pruitt S., Rai A., Espinoza N., Valladares A., Spinard E., Silva E., Velazquez-Salinas L., Gladue D.P. (2022). ASFV Gene A151R Is Involved in the Process of Virulence in Domestic Swine. Viruses.

[35] Lewis T., Zsak L., Burrage T.G., Lu Z., Kutish G.F., Neilan J.G., Rock D.L. (2000). An African swine fever virus ERV1-ALR homologue, 9GL, affects virion maturation and viral growth in macrophages and viral virulence in swine. J. Virol..

[36] Afonso C.L., Zsak L., Carrillo C., Borca M.V., Rock D.L. (1998). African swine fever virus NL gene is not required for virus virulence. J. Gen. Virol..

[37] Zsak L., Lu Z., Kutish G.F., Neilan J.G., Rock D.L. (1996). An African swine fever virus virulence-associated gene NL-S with similarity to the herpes simplex virus ICP34.5 gene. J. Virol..

[38] Borca M.V., Carrillo C., Zsak L., Laegreid W.W., Kutish G.F., Neilan J.G., Burrage T.G., Rock D.L. (1998). Deletion of a CD2-like gene, 8-DR, from African swine fever virus affects viral infection in domestic swine. J. Virol..

[39] Li D., Liu Y., Qi X., Wen Y., Li P., Ma Z., Liu Y., Zheng H., Liu Z. (2021). African Swine Fever Virus MGF-110-9L-deficient Mutant Has Attenuated Virulence in Pigs. Virol. Sin..

[40] Borca M.V., Ramirez-Medina E., Silva E., Vuono E., Rai A., Pruitt S., Espinoza N., Velazquez-Salinas L., Gay C.G., Gladue D.P. (2021). ASFV-G-I177L as an Effective Oral Nasal Vaccine against the Eurasia Strain of Africa Swine Fever. Viruses.

[41] Zhang J., Zhang Y., Chen T., Yang J., Yue H., Wang L., Zhou X., Qi Y., Han X., Ke J. (2021). Deletion of the L7L-L11L Genes Attenuates ASFV and Induces Protection against Homologous Challenge. Viruses.

[42] Gladue D.P., Borca M.V. (2022). Recombinant ASF Live Attenuated Virus Strains as Experimental Vaccine Candidates. Viruses.

[43] Wen X., He X., Zhang X., Zhang X., Liu L., Guan Y., Zhang Y., Bu Z. (2019). Genome sequences derived from pig and dried blood pig feed samples provide important insights into the transmission of African swine fever virus in China in 2018. Emerg. Microbes Infect..

[44] Tran X.H., Phuong L.T.T., Huy N.Q., Thuy D.T., Nguyen V.D., Quang P.H., Ngôn Q.V., Rai A., Gay C.G., Gladue D.P. (2022). Evaluation of the Safety Profile of the ASFV Vaccine Candidate ASFV-G-&Delta;I177L. Viruses.

[45] Zhang Y., Ke J., Zhang J., Yang J., Yue H., Zhou X., Qi Y., Zhu R., Miao F., Li Q. (2021). African Swine Fever Virus Bearing an I226R Gene Deletion Elicits Robust Immunity in Pigs to African Swine Fever. J. Virol..

[46] Ran Y., Li D., Xiong M.G., Liu H.N., Feng T., Shi Z.W., Li Y.H., Wu H.N., Wang S.Y., Zheng H.X. (2022). African swine fever virus I267L acts as an important virulence factor by inhibiting RNA polymerase III-RIG-I-mediated innate immunity. PLoS Pathog..

[47] Li D., Zhang J., Yang W., Li P., Ru Y., Kang W., Li L., Ran Y., Zheng H. (2021). African swine fever virus protein MGF-505-7R promotes virulence and pathogenesis by inhibiting JAK1- and JAK2-mediated signaling. J. Biol. Chem..

